# Effect of chronic estradiol plus progesterone treatment on experimental arterial and venous thrombosis in mouse

**DOI:** 10.1371/journal.pone.0177043

**Published:** 2017-05-09

**Authors:** Marie-Cécile Valéra, Emmanuelle Noirrit-Esclassan, Marion Dupuis, Melissa Buscato, Alexia Vinel, Maeva Guillaume, Anne Briaux, Cédric Garcia, Thibaut Benoit, Olivier Lairez, Coralie Fontaine, Bernard Payrastre, Jean-François Arnal

**Affiliations:** 1 Inserm, U1048 and Université Toulouse III, I2MC, Toulouse, France; 2 Faculté de Chirurgie Dentaire, Université de Toulouse III, Toulouse, France; 3 Laboratoire d’Hématologie, CHU de Toulouse, Toulouse, France; 4 Service d’urologie, CHU de Toulouse, Toulouse, France; Ludwig-Maximilians-Universitat Munchen, GERMANY

## Abstract

Postmenopausal hormone replacement therapy (HRT) with estrogen plus progestogens is the first line therapy to treat menopausal symptoms. The progestogen is added to estrogen to reduce or eliminate the excess risk of endometrial cancer due to the unopposed effect of estrogen. Whereas progestin clearly opposes the proliferative and deleterious long-term actions of estrogen on the endometrium, the interference of progestin on the other estrogen action remains unclear. We previously reported that chronic subcutaneous 17α-estradiol (E2) in mice decreases platelet responsiveness, prolongs the tail-bleeding time and protects against acute thromboembolism. Here, we report the tissue-specific interference of progesterone (P4) on the action of E2 in ovariectomized mice. We first confirm that, in our experimental conditions, P4 attenuates the proliferative action of E2 on the uterus and the effects of E2 on vagina weight and lubrication. We then studied the effect of E2 combined with P4 on hemostasis and thrombosis *in vivo* in mice and found that P4 did not interfere with the main actions of E2 on platelets, bleeding time and arterial and venous thrombosis. Thus, whereas activation of progesterone receptor interferes with the action of E2 on its classic sex targets, P4 appears to have minimal effect on the hemostasis and thrombosis actions of E2, supporting the prominent role of estrogens and the accessory role of natural progestin on the extra-reproductive cells and tissues involved in thrombosis.

## Introduction

Postmenopausal hormone replacement therapy (HRT) with estrogen alone or estrogen plus progestogens is the first line therapy to treat menopausal symptoms such as hot flushes, vaginal atrophy and insomnia. Progestogens include both progesterone (P4) and synthetic progestins derived from progesterone (pregnanes and 19-norpregnanes) or from testosterone (19-nortestosterones). P4 is an endogenous steroid hormone produced from cholesterol in the ovaries by the corpus luteum during the luteal phase of the menstrual cycle and in the adrenal glands. This hormone is involved in the function of female reproductive tissues, embryo implantation, maintenance of pregnancy and embryogenesis [1]. Progestogens produce their physiological and biological effects by interacting with intracellular progesterone receptors A (PR-A) and B (PR-B). PRs, like other sex-steroid hormone receptors, belong to the nuclear receptor superfamily of transcription factors and regulate gene expression following hormone binding [2,3]. PRs are expressed in many cells of female reproductive tissues (uterus, ovaries, mammary glands) but they also have effects on the vascular cells, where PR expression is induced by estrogen [4]. The effects of progestogens are related to the interactions not only with PRs but also with other steroid receptors such as androgen receptor, glucocorticoid receptor and mineralocorticoid receptor [3].

Harmful effects of HRT include increased risk of breast cancer, coronary heart disease, stroke and thromboembolic disorders [5]. Venous thromboembolism (VTE), including deep venous thrombosis (DVT) and pulmonary embolism (PE), accounts for about one third of all potentially fatal cardiovascular events in postmenopausal women using HRT [6]. The impact of the route of estrogen administration on thromboembolic events has been investigated [7–9]. Overall, these observational studies demonstrated an increased risk of VTE in users of oral postmenopausal estrogen, but not with transdermal E2. This increased VTE risk is attributed to the impact of the route of estrogen administration on blood coagulation through the hepatic first pass.

Progestogens demonstrate a strong anti-oestrogenic activity in the endometrium and the addition of a progestogen to estrogens therapy among postmenopausal women with an intact uterus is required for preventing the elevated risk of estrogen-induced endometrial hyperplasia and adenocarcinoma [10]. The effects of the type of progestogens added on thromboembolic disorders were recently detailed in a meta-analysis based on observational studies [11]-: there is an increased risk of VTE (by about 50%) in women using estrogens and progestogens as compared with users of oral estrogens alone. Practices regarding the type of progestogens used in HRT are different in America and in Europe. In fact, a variety of progestogens are used in European countries [7,12,13] but in the USA, medroxyprogesterone acetate (MPA) is used almost exclusively. In this context, the type of progestogen has recently emerged as an important determinant of the VTE risk among HRT users [12,14].

We recently attempted to investigate the complexity of sex hormones actions on hemostasis and thrombosis in mouse. 17α-estradiol (E2) is a steroid hormone made from cholesterol and is the most potent form of mammalian estrogenic steroids. We reported that chronic E2 treatment, administered subcutaneously, decreased platelet responsiveness, increased tail-bleeding times and protected animals against collagen/epinephrine-induced thromboembolism and carotid artery thrombosis in comparison to ovariectomized or sham-operated mice [15]. This effect is mediated by hematopoietic estrogen receptor (ER) alpha since it is completely abolished using chimeric hematopoietic mice harboring a selective deletion of estrogen receptors ERα [15]. The aim of the present study was to define the impact of a chronic subcutaneous administration of E2+P4 on thromboembolism and on carotid artery and inferior vena cava thrombosis in mouse.

## Materials and methods

### Materials

Collagen reagent HORM® (type I fibrils from equine tendon) suspension was purchased from Takeda, epinephrine from Sigma-Aldrich and FeCl_3_ from Mallinckrodt Chemical. Anti–GPIbα was from Emfret Analytics.

### Mice

Female C57BL/6J mice were purchased from Charles River. All procedures were performed in accordance with the principles established by the National Institute of Medical Research and were approved by the local Ethical Committee of Animal Care. Prepubertal mice (4 weeks of age) were anesthetized by intraperitoneal injection of ketamine (25 mg/kg) and xylazine (10 mg/kg) and ovariectomized to avoid endogenous estrogens production. Treatments were started or not (OVX mice) approximately 2 weeks after ovariectomy to allow for complete recovery from surgery. Ovariectomized mice were implanted subcutaneously with pellets releasing E2 (0.1 mg, i.e 80 μg/kg/day, Innovative Research of America), P4 (10 mg, Innovative Research of America) or E2 and P4 (E2+P4). Mice were euthanized after a 3-week treatment period at 9–10 weeks of age, which correspond to adult mice. Blood was collected from the inferior vena cava and uteri and vagina were collected and weighed. Bone marrow cells were flushed from femurs, resuspended in ACK lysis buffer (0.15M NH4Cl; 1 mM KHCO3; 0.1 mM Na2EDTA; pH7.3) to lyse mature erythrocytes and counted with a Beckman Coulter Counter.

### Histological analysis

Paraffin-embedded transverse sections (4 μm) from formalin-fixed uterine or vagina specimens were stained as previously described [16] with anti–Ki-67 antigen (RM-9106; Thermo-scientific). Sections were examined after numerisation using NanoZoomer Digital Pathology®. To examine the proliferative effect of each treatment, the ratio of Ki-67–positive epithelial/total cells number on the entire luminal epithelium from each uterine or vaginal section was evaluated. The luminal epithelial height (LEH) was measured from the basal membrane to the apical surface as previously described for the uterus [16,17]. The values are the mean of 10 measurements in each transverse uterus or vaginal section.

### Cervical vaginal lubrication (CVS)

Under anaesthesia, stimulation of the vagina was realized by five strokes within 5 seconds against the cervix by a 3French (1.0 mm wide) Teflon-coated probe inserted into the vagina lumen (3Fr occlusion balloon catheter, Coloplast; Rosny-sous-Bois, France) as previously described [18]. One minute after stimulation, a pre-weighed absorbent paper strip was inserted into the vagina for 10 seconds. Vaginal lubricate volume was estimated from the difference in weights (mg) after and before insertion (Mettler AC 100 analytical balance, Mettler Toledo, France).

### Tail-bleeding time

After mice anesthesia, we measured bleeding time by 3-mm tail-tip transection. Blood drops were removed every 15 seconds with the use of a paper filter. If bleeding did not recur within 30 seconds of cessation, it was considered stopped. Experiments were terminated after 30 minutes if no cessation of blood flow occurred.

### Thromboembolism and echocardiography

Acute systemic vascular thromboembolism was induced by injecting a mixture of collagen (0.4 mg/kg) and epinephrine (60 μg/kg) into the right jugular vein of anesthetized mice. Mice were euthanized 10 minutes after injection of the mixture for histology analysis. To measure the resistance to thromboembolism, the percentage of mortality at 10 minutes was determined. Transthoracic echocardiographies were performed with a General Electric Vivid 7® (*GE Medical System*, Milwaukee, USA) equipped with a 13-MHz linear probe. The animals were placed in the supine position. Numeric images of the heart were obtained before injection, at injection and 5 and 10 minutes after, in parasternal short-axis views standardized by including the mild-papillary muscles. All mice were assessed but the measurements were stopped once the animal died. Two-dimensional echocardiographic measurements were performed with the cine-loop feature to retrospectively catch true end-diastolic phase that was defined as the phase in which the largest left ventricular (LV) cavity size was obtained. Endocardial LV (red line, S1 Fig) and right ventricular (blue line, S1 Fig) end-diastolic areas were obtained by hand-tracings of LV endocardial and right ventricular endocardial contours, respectively. The LV sphericity index was calculated on the same end-diastolic frame by the ratio between the cross-sectional diameter of the LV at the point of insertion of the RV wall (D1, S1 Fig) and the LV diameter measured at right angles (D2, S1 Fig). This index was used as an indicator of RV pressure overload. All measurements were averaged on 3 different cardiac cycles and analyzed by a single observer who was blinded to the treatment status of the animals.

### Inferior vena cava thrombosis

Mice were anesthetized and after laparotomy, intestines were exteriorized and sterile saline was applied during the whole procedure to prevent drying. After gentle separation from aorta, inferior vena cava (IVC) was ligated by a 8.0 polypropylene suture immediately below the renal veins to obtain complete blood stasis as previously described [19]. Mice were euthanized after 24 hours. The thrombi formed in the IVC were carefully dissected, weighted and removed for formalin fixation and paraffin embedding immunohistochemistry.

### Carotid artery thrombosis

We used the Vevo2100 high-frequency ultrasound system (HFUS) (Visualsonics, Toronto, Canada) to monitor thrombus formation in the right carotid artery of mice. This micro imaging system consists of a single element probe of 18–38 MHz of frequency. Heart rate of the animal was monitored and kept at 400/500 beats per min. Temperature was monitored using a rectal probe and regulated with a heating pad. The carotid was dissected free from surrounding tissues. FeCl_3_ was used to induce vascular injury. A 1 × 4-mm strip of paper saturated with 7% FeCl_3_ solution was applied to the adventitial surface of the left carotid for 3 minutes then removed. Warm ultrasound transmission gel was applied to enable visualization and optimize image quality.

### Induced thrombocytopenia

Thrombocytopenia was induced after a 3 weeks treatment by intraperitoneal injection of anti-mouse GPIbα antibody (2 μg/g body weight). Blood samples were collected before injection and then at D2, D4, D7, D9, D12 and D15 after injection by submandibular blood collection. Platelet counts were measured using a Micros60 (Horiba ABX Diagnostics).

### Analysis of mRNA levels by qPCR

Aortas were homogenized using a Precellys tissue homogenizer (Bertin Technology, Cedex, France), and total RNA from tissues was prepared using TRIzol reagent (Invitrogen, Carlsbad, CA). A total of 500ng was reverse transcribed for 10 minutes at 25°C and for 2 hours at 37°C in a 20-μL final volume using the High Capacity cDNA Reverse Transcriptase Kit (Applied Biosystems, Villebon sur Yvette, France). Quantitative real-time PCRs (qPCRs) were performed on the StepOne instrument (Applied Biosystems). Primers were validated by testing PCR efficiency using standard curves (95% ≤ efficiency ≤ 105%). Gene expression was quantified using the comparative C_T_ method; hypoxanthine guanine phosphoribosyl transferase 1 (HPRT) was used as a reference.

### Coagulation tests and dosage of coagulation factors

Prothrombin time (PT) and activated partial thromboplastin time (aPTT) were semi automatically measured on a START4 (Diagnostica Stago) using the STA-Neoplastine kit (Diagnostica Stago) for PT, the STA-C.K. Prest kit (Diagnostica Stago) for aPTT and normal human plasma (SHP, Siemens) as a standard. Fibrinogen measured with STA-Fibriprest kit (Diagnostica Stago). The end point was the time to clot formation as measured automatically. We measured the functional activity of FII, FV, FVII, FVIII, FIX, FX, and FXI. The assays were modified from human clinical assays by mixing mouse plasma with factor-deficient human plasma (Diagnostica Stago). Standard curves for each assay were generated using normal human plasma, and percent activity of each factor was calculated. All activities were then normalized to a reference of 100% as previously published [15].

### Statistical analysis

Results are expressed as mean ± SD or SEM, as indicated. Statistical analyses were performed using graph pad. To test the respective roles of each treatment, a one‐way ANOVA was performed and a Bonferroni's multiple comparison test. * P<0.05, ** P <0.01, *** P<0.001.

## Results

### Effect of E2 and P4 on the uteri and vagina and at the level of gene expression in the aorta

**Fig 1:**
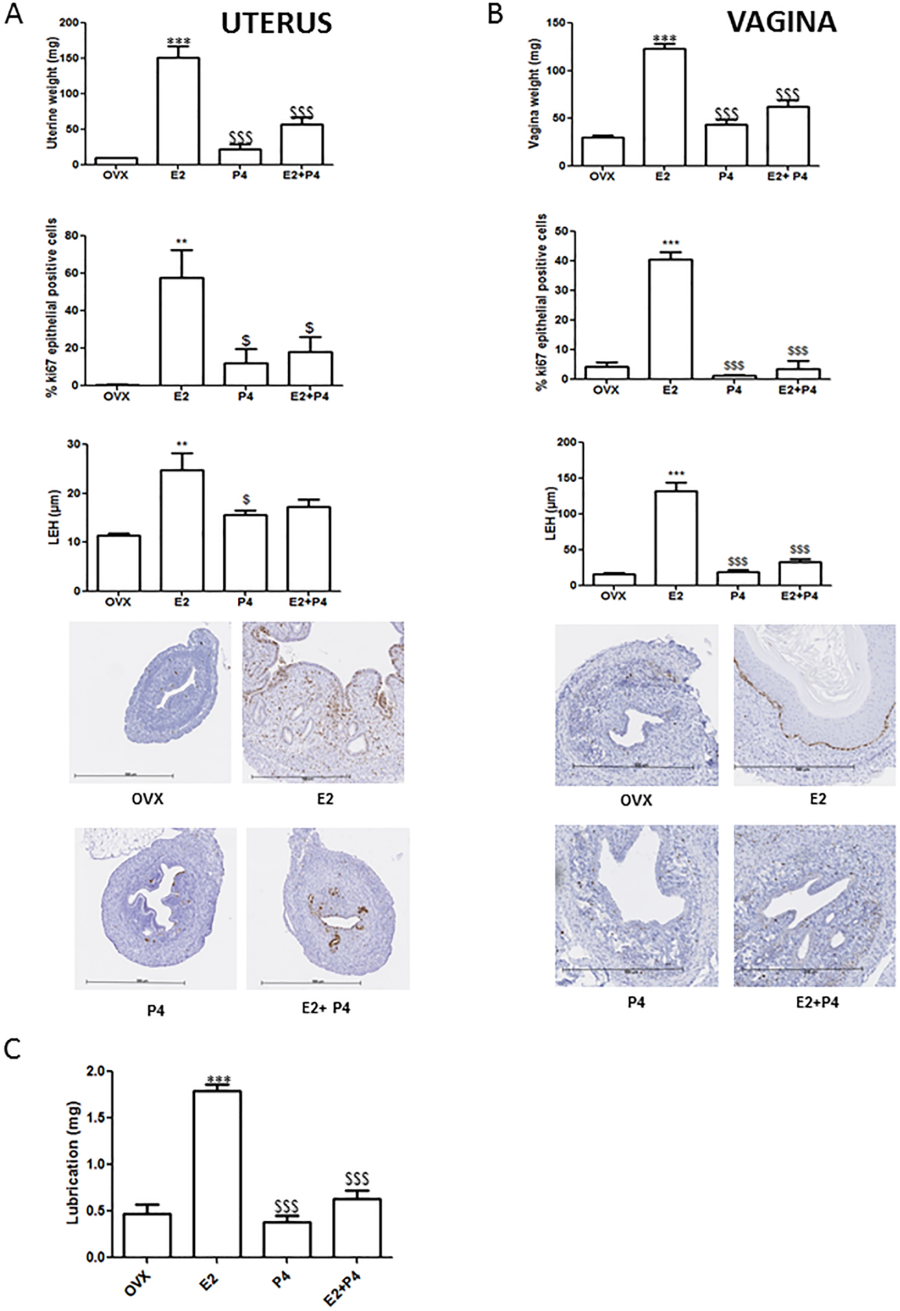
Effects of a chronic administration of E2, P4 and combined treatment on the uteri and vagina of mice. **(A)** Uterine weights, epithelial proliferation and luminal epithelial height after the different treatments. Representative sections from uterus stained with Ki-67 antigen. **(B)** Vagina weights, epithelial proliferation and luminal epithelial height after the different treatments. Representative sections from vagina stained with Ki-67 antigen are shown. **(C)** Vaginal lubrication (mg). Data are presented as mean ± SD. To test the respective roles of each treatment, a one-way ANOVA was performed and a Bonferroni’s multiple comparison test. * t test vs OVX. $ t test vs E2.

First, we aim to evaluate the effects of P4 on E2 response *in vivo* in both reproductive and extra-reproductive tissues. The relative effect of estrogen and P4 was evaluated through the use of hormone pellets (E2 0.1 mg and P4 10 mg) on ovariectomized mice. Treatment of mice with E2 or P4 alone or combined revealed no difference in body weight between ovariectomized untreated mice (mean = 20.9g ±1.6, n = 9), E2-treated mice (21.7g ±1.6, n = 13), P4-treated mice (23.3g ±1.7, n = 10) and E2+P4-treated mice (22.1g ±1.2, n = 16). As expected, uterine weights, epithelial proliferation and luminal epithelial length were greatly increased following E2 treatment compared to ovariectomized and to P4-treated mice (Fig 1A). By contrast, uterine weights from E2+P4-treated mice were significantly lower than those of E2-treated mice, indicating the efficacy of the treatment. In addition and as evaluated by immunohistochemical detection of Ki-67 antigen, the percentage of luminal epithelial Ki-67 positive cells was significantly decreased in E2+P4-treated mice compared to E2-treated mice (Fig 1A). Following E2 treatment, the uterine stroma significantly decreased in terms of cell density and presented loosely arranged oval fibroblasts, glands with cystic dilatation and neutrophilic infiltration. Conversely, uterine stroma from mice receiving E2 and P4 was compact and dense without mitotic activity and the luminal epithelium was composed of cuboidal, non-proliferating cells with a high nuclear/cytoplasmic ratio.

In comparison to ovariectomized and to P4-treated mice, vagina weights, epithelial proliferation and luminal epithelial length were increased following E2 treatment (Fig 1B). Vagina weights from E2+P4-treated mice were lower than those of E2-treated mice, indicating a antagonist effect of P4 on E2 vaginal response (Fig 1B). In addition, vaginal lubrication after cervical vaginal stimulation was significantly decreased in E2+P4 mice compared to E2-treated mice (Fig 1C). In order to evaluate the effects of P4 association to E2 in extra reproductive tissues, we also analyzed ERα target gene expression in the aorta (S2 Fig). As expected, mRNA of Grem2 and Cam2kb were strongly induced after E_2_ treatment in the aorta whereas Shisa expression was decreased [20]. By contrast, P4 had no impact on expression of these genes in ovariectomized or in E2-treated mice, demonstrating that in the vessel P4 does not antagonize transcriptional response induced by E2. Expression of ERα or PR mRNA level was not impacted by these different treatments.

Overall, E2+P4 treatment induced both structural and functional changes on the vagina and uteri but had no impact on the level of gene expression in the aorta, indicating tissue specific action of P4 and E2 –mediated effect.

### E2+P4 treatment increases tail-bleeding time

**Fig 2:**
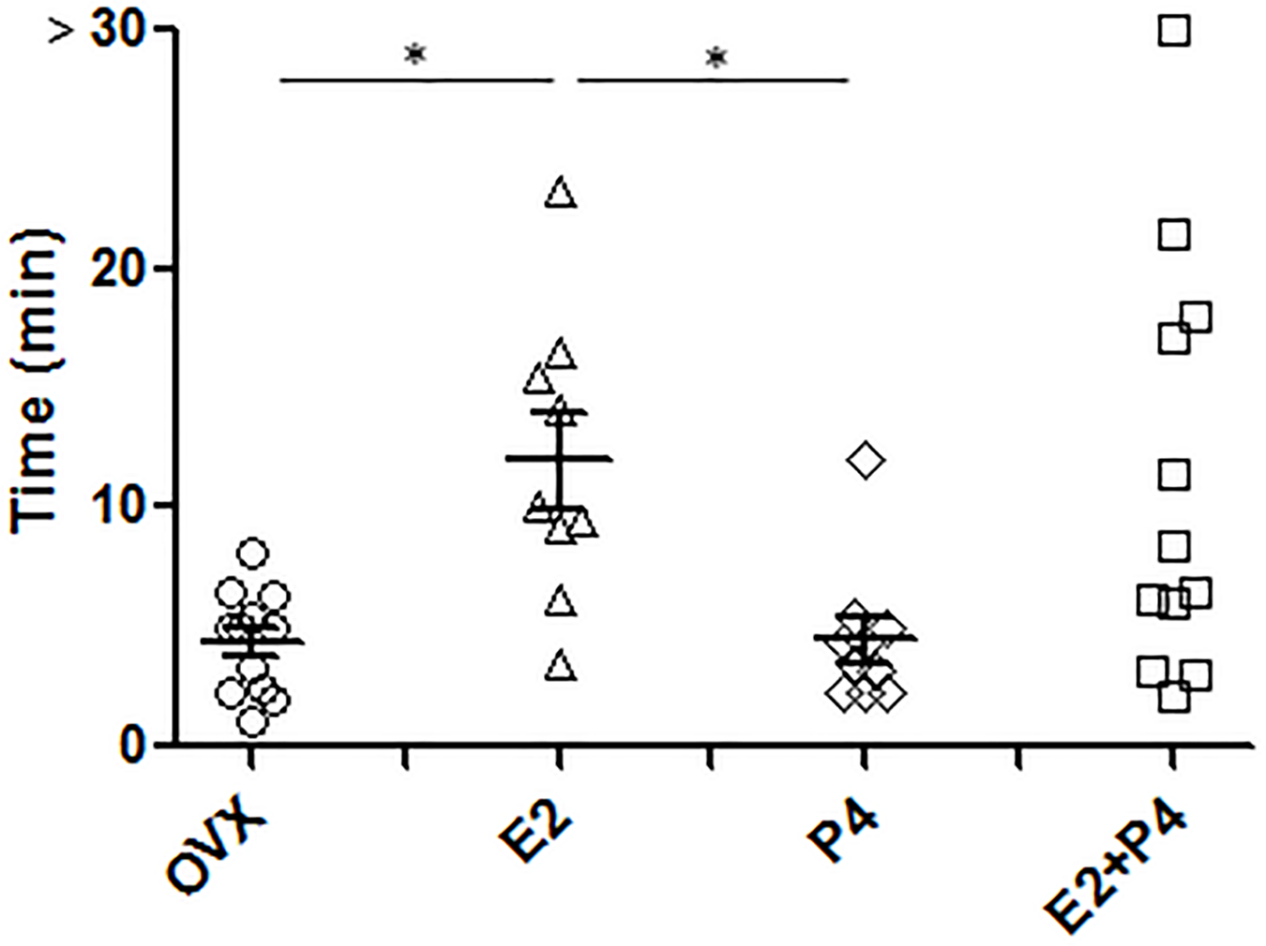
Increased bleeding time in E2, P4 and E2+P4-treated mice. The tail-bleeding times of untreated (OVX, n = 12), E2 (n = 9)-, P4 (n = 10)- and E2+P4 (n = 12)- treated mice were measured as described in Methods. Each point represents 1 individual

Primary hemostasis was next analyzed *in vivo* by measuring the tail-bleeding time (Fig 2). The tail-bleeding times of untreated ovariectomized mice and P4-treated mice were normal (4.4 minutes, ±2.1, n = 12 and 4.4 minutes±2.8, n = 10 respectively) while, as expected [15], E2 treatment prolonged the tail-bleeding time. E2+P4 co-treatment also increased the tail-bleeding time with a bleeding prolonged above 10 minutes in 5 out of 12 mice analyzed (Fig 2) demonstrating that P4 does not interfere on this E2 effect.

### E2+P4 treatment protects mice from venous and arterial thrombosis

**Fig 3:**
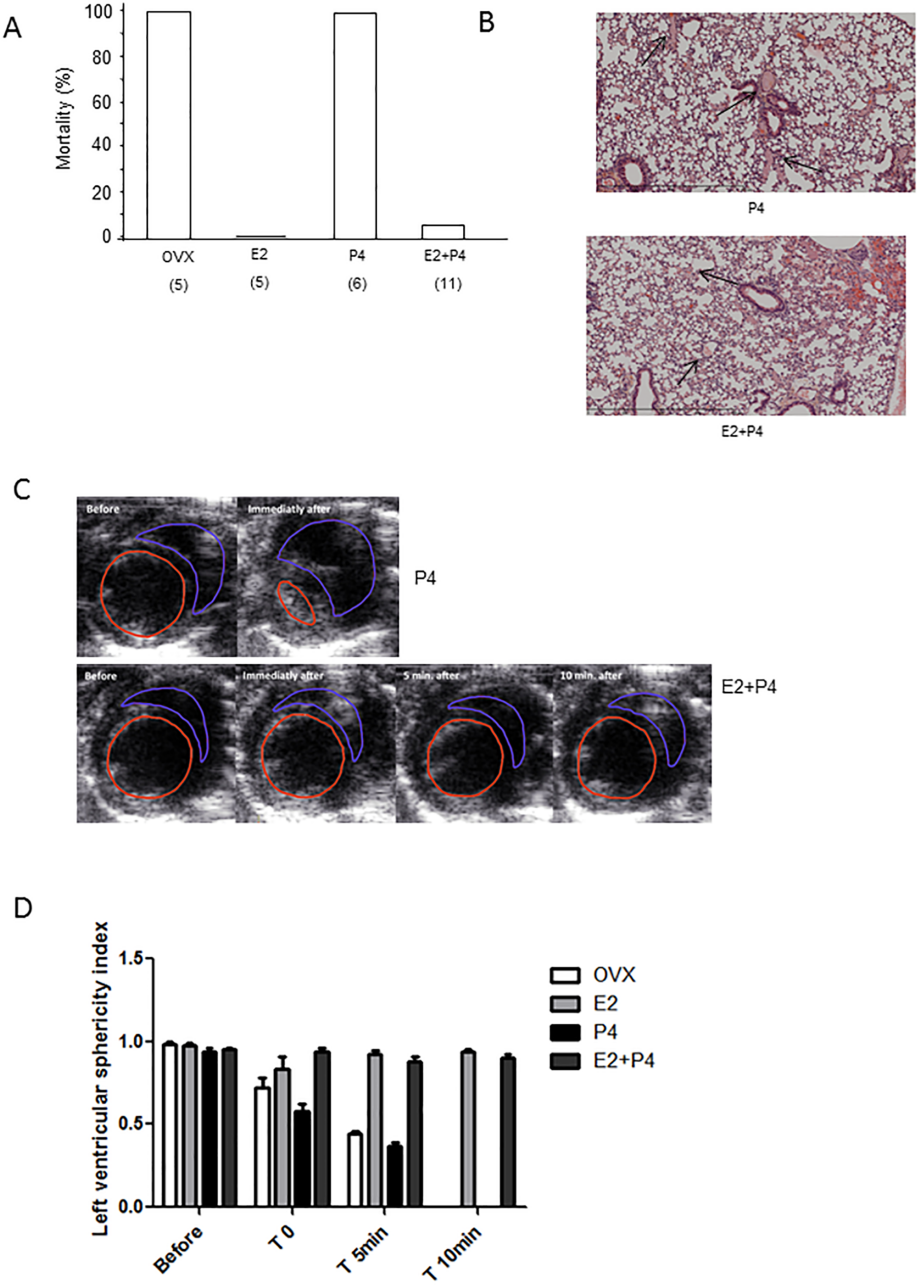
E2+P4 treatment protects mice against thromboembolism. Thromboembolism was induced by injection of a collagen (0.4 mg/kg) and epinephrine (60 μg/kg) mixture into the jugular vein. **(A)** All ovariectomized mice (OVX) died within 5 minutes. E2- and E2+P4- treated mice were protected from thromboembolism. **(B)** Representative sections of hematoxylin-eosin–stained lungs from P4- and E2+P4 -treated mouse are shown. Arrows point to thrombi in the pulmonary vasculature of OVX and treated mice. Original magnification ×250. **(C)** Representative images of the heart of P4- and E2+P4 -treated mouse at the indicate times are shown. The left ventricular (LV) sphericity index was calculated by the ratio between the cross-sectional diameter of the LV at the point of insertion of the right ventricular wall (D1) and the LV diameter measured at right angles (D2) **(D)** Quantification of the left ventricular sphericity index measured before, at time of induction of embolism and 5 and 10 minutes after.

While all ovariectomized untreated and P4-treated mice died from thromboembolism within 5 minutes of injection of collagen/epinephrine mixture into the jugular vein, all E2 -treated mice (n = 5) were still alive 10 minutes after administration of the pro-thrombotic mixture (Fig 3A). Only 1 out of 11 mice died within 10 minutes of injection when they received E2 and P4 co-treatment (Fig 3A). Histological analysis of mouse lungs harvested 10 minutes after the injection showed marked protection from thrombi in vessels of E2- and E2+P4-treated mice compared to control mice (Fig 3B). Using transthoracic echocardiography, we measured the sphericity index as an indicator of right ventricular pressure overload (Fig 3C and 3D). We found that left ventricular geometry as assessed by the sphericity index (SI) was altered in untreated ovariectomized and P4-treated mice (at 5 minutes, SI = 0.4±0.01 and SI = 0.4±0.05, respectively). Conversely, mice protected from collagen/epinephrine induced thromboembolism showed normal left ventricular morphology (at 5 minutes, SI = 0.9±0.06 for E2-treated mice and SI = 0.9±0.1 for E2+P4-treated mice) (Fig 3D).

**Fig 4:**
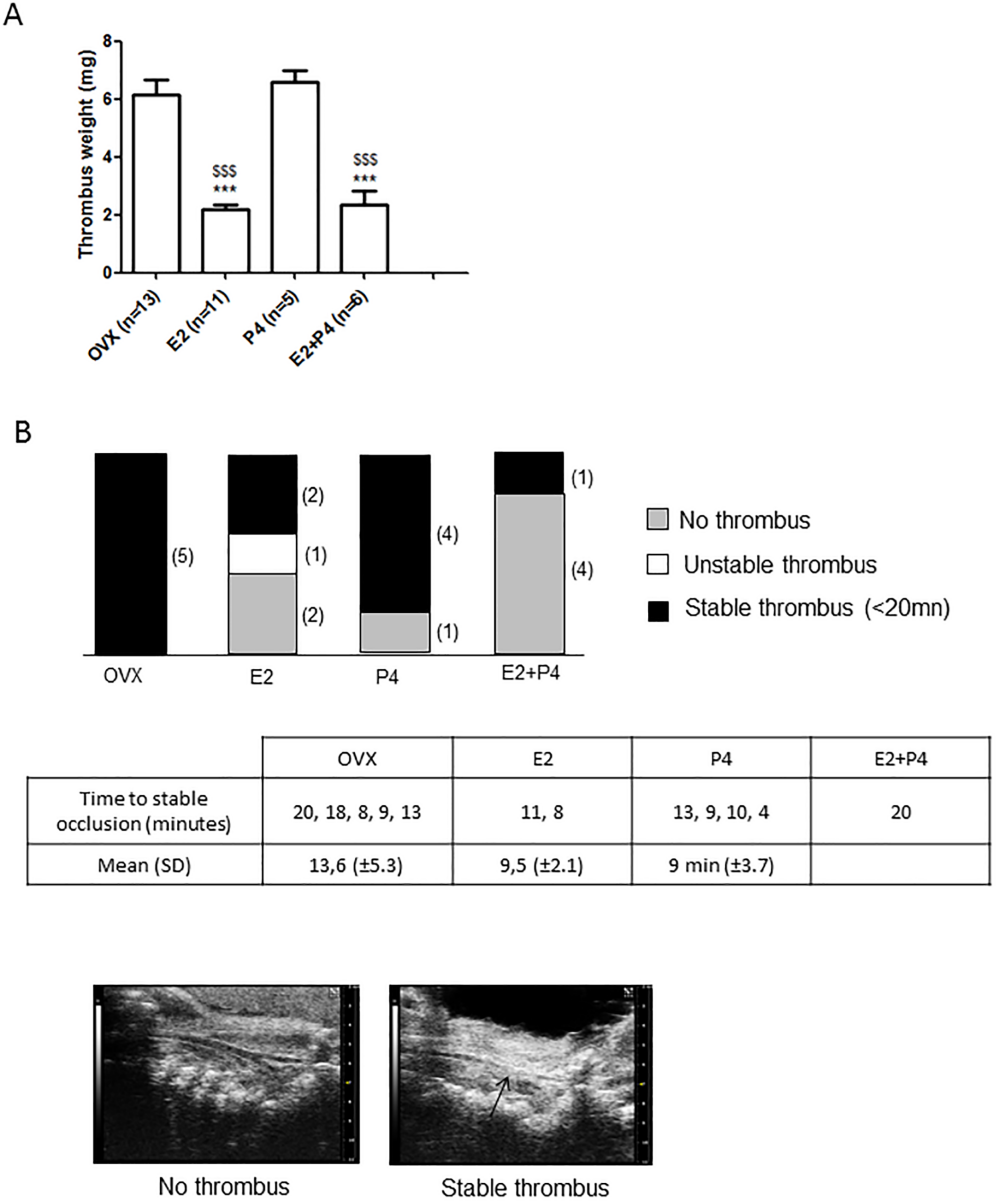
Inferior vena cava stasis in OVX and E2-, P4- and E2+P4- treated mice. **(A)** 24 hours after the induction of the stasis in the inferior vena cava by ligation, mice were killed and thrombi were harvested. The mean weight of the thrombi is presented ± SD. * t test vs OVX, $ t test vs E2. **(B)** Following ferric chloride injury of the carotid artery, the thrombus formed was visualized by high-frequency ultrasound after. Longitudinal views of a mouse without thrombus and with a stable thrombus are shown.

We then tested the impact of E2+P4 in a mouse model of venous thrombosis induced by inferior vena cava (IVC) stasis following total ligation [21]. In this model, thrombosis results from blood flow arrest leading to endothelial cell activation and subsequent, progressive and extensive thrombosis. The thrombus mass was quantified 24 hours after stasis induction. As shown in Fig 4A, compared to untreated ovariectomized and P4-treated mice, E2-as well as E2+P4-treated mice developed significantly smaller thrombi, as assessed by measurement of the mean thrombus weight which was 6.1 mg (±1.9, n = 13), 6.6 mg (±0.9, n = 5), 2.2 (±0.6, n = 11) and 2.3 mg (±1.2, n = 6), respectively.

We then tested the effect of E2+P4 in preventing occlusive thrombus formation in the right carotid artery following FeCl_3_-induced carotid injury (Fig 4B). The time-to-artery occlusion was determined by echography and a complete and stable occlusion was observed within 20 minutes in all untreated ovariectomized and 80% of P4-treated mice (Fig 4B). The mean time-to-occlusion was 13.6 min (±5.3, n = 5) and 9 min (±3.7, n = 4) for ovariectomized and P4-treated mice, respectively. In contrast, E2-treated mice showed a significant resistance. Indeed, 40% of the mice exhibited a complete resistance to occlusion, 20% developed unstable occlusions and 40% a complete and stable occlusion. The time-to-occlusion was 11 and 8 minutes for these 2 E2 treated mice. Eighty % of E2+P4-treated mice exhibited a complete resistance to occlusion. One mouse developed a stable thrombus at 20 minutes.

Taken together, these data suggest that P4 treatment did not interfere with the protective effect of E2-treatment on arterial and venous thrombosis in mice.

### Hematologic parameters

**Fig 5:**
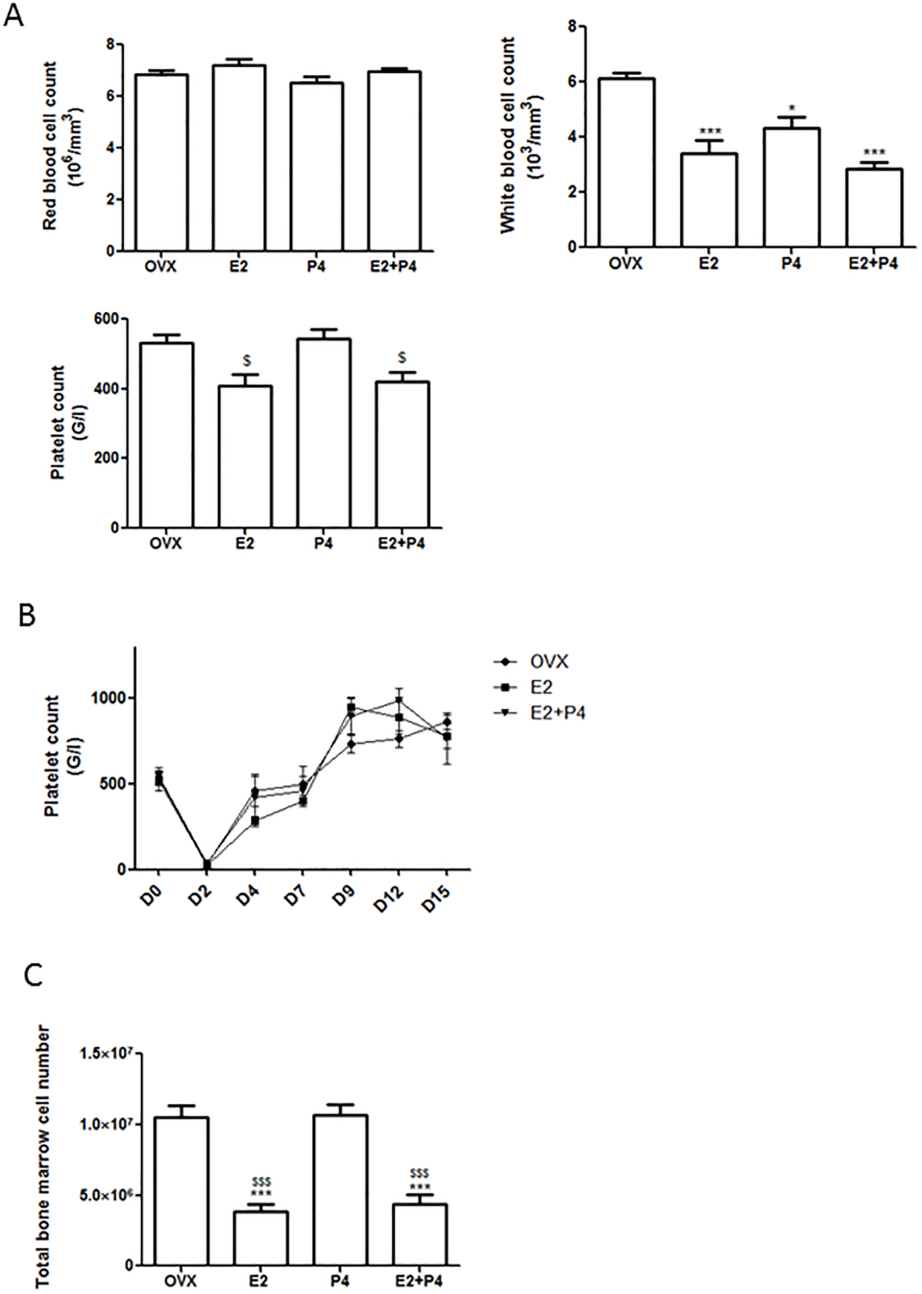
Hematologic parameters. **(A)** Blood cells counts in ovariectomized (OVX), E2, P4- and E2+P4-treated mice. OVX (n = 5), +E2 (n = 5), P4 (n = 5), E2+P4 (n = 5). **(B)** Platelet count recovery after immune thrombocytopenia. Thrombocytopenia was induced after 3 weeks of treatment by intraperitoneal injection of anti-mouse GPIbα antibody. Blood samples were collected before injection (D = 0) and 2, 4, 7, 9, 12 and 15 days after. OVX (n = 5), E2 (n = 5), E2+P4 (n = 5). **(C)** Total bone marrow cell numbers between OVX, E2-, P4- and E2+P4- treated mice. Bone marrow was flushed from 2 femurs and total bone marrow cell numbers was obtained using a Beckman Coulter Counter. OVX (n = 8), E2 (n = 5), P4 (n = 5), E2+P4 (n = 6). Mean ± SD. To test the respective roles of each treatment, a one-way ANOVA was performed and a Bonferroni’s multiple comparison test. * t test vs OVX. $ t test vs P4.

The effect of estrogen and P4 on blood cell count and coagulation was then evaluated. Red blood cells counts were not affected by the different treatments. In comparison to ovariectomized mice, white cell count was decreased in E2-treated mice, as previously described [22], and in mice treated with P4 and E2+P4 (Fig 5A). In E2+P4 and E2-treated mice, platelet count was not significantly affected in comparison to ovariectomized mice (Fig 5A). Consistent with this finding, the platelet count recovery at day 15 after immune-induced thrombocytopenia was similar in all groups: platelet production did not seem to be impacted by the treatments (Fig 5B). In addition, total bone marrow cell number was the same in bone marrow flushes from femurs of OVX and P4-treated mice but was decreased in E2 and E2+P4-treated mice compared to untreated and P4-treated mice (Fig 5C).

Standard coagulation tests as well as dosage of fibrinogen and coagulation factors were also performed. In comparison to untreated ovariectomized mice, E2+P4-treated mice displayed an increased level of Factor IX. The different treatments had no impact on prothrombin time, activated partial thromboplastin time and on the levels of plasma fibrinogen and others coagulation factors compared to control mice (Table 1). Overall these data showed that, compared to vehicle, E2+P4 administered chronically had no impact on coagulation factors. Thus, E2+P4 effects on primary hemostasis and thrombosis were not due to functional deficiencies in coagulation or modification of the platelet count.

**Table 1:** Hematological parameters. Coagulation tests (prothrombin time (PT) and activated partial thromboplastin time (APPT)), fibrinogen and coagulation factors levels in ovariectomized (OVX) and treated mice with E2, P4 and E2+P4. Means ± SD. ** p<0.001 indicates significantly different from OVX.

	OVX	E2	P4	E2+P4
**PT**	101.8 +/- 3.2	97.0 +/- 10.1	100.4 +/- 11.3	93.0 +/- 9.8
**APTT**	48.7 +/- 19.6	47.8 +/- 20.6	49.8 +/- 20.1	47.0 +/- 7.2
**Fibrinogen**	1.5 +/- 0.1	1.98+/- 0.4	1.4 +/- 0.1	1.7 +/- 0.3
**Factor II**	41.2 +/- 3.7	45.0 +/- 4.5	40.0 +/- 4.6	44.2 +/- 4.8
**Factor V**	293.0 +/- 11.1	296.4 +/- 10.1	260.6 +/- 23.5	276.4 +/- 15.9
**Factor VII**	201.8 +/- 11.6	222.8 +/- 10.4	216.6 +/- 7.1	187.6+/- 14.5
**Factor X**	100.2 +/- 5.2	107.6 +/- 6.9	100.6 +/- 5.5	101.8 +/- 6.1
**Factor VIII**	207.6 +/- 31.5	256.6 +/- 25.2	170.2 +/- 42.6	207.6 +/- 51.7
**Factor IX**	29.6 +/- 5.4	36.4 +/- 5.6	32.0 +/- 6.6	50.0 +/- 9.9 **

## Discussion

In the present work, we explored the impact of a chronic treatment of E2+P4 on hemostasis and on thromboembolism and on carotid artery and inferior vena cava thrombosis in mouse. The addition of progestogens is required among postmenopausal women with an intact uterus in order to prevent the elevated risk of estrogen-induced endometrial hyperplasia and adenocarcinoma. Estrogen/progesterone ratio typically administered is 10:1 to 20:1 in human HRT and 10:1 to 100:1 in mice studies [23,24]. The ratio used in the current study was 100:1 and the doses selected were sufficient after a 3 weeks chronic treatment in ovariectomized mice to induce structural and functional changes not only in the uterus but also the vagina.

We highlighted here for the first time that, in mice, E2 combined with P4 led to increased tail-bleeding time, resistance to collagen/epinephrine-induced thromboembolism and protection against venous and arterial thrombosis. Echocardiography showed a normal left ventricular function in mice treated with E2 alone or combined with P4 and protected against thromboembolism. Conversely, mice developing thromboembolism displayed severe and lethal ventricular dysfunction, characterized by a major decrease of left ventricular size associated to a marked increase in volume of the right ventricular. We further confirmed the protection against thrombosis in E2 and E2+P4-treated mice in two well-described models: i) venous thrombosis induced by IVC stasis following ligation [21] and ii) thrombus formation in the right carotid artery following FeCl_3_-induced injury [25]. Remarkably, protection conferred by the two treatments occurred in both arterial and venous vascular thrombosis models. Consistently, we showed here that E2 combined with P4 protected young adult mice from thrombosis independently of functional deficiencies in coagulation or defects in platelet count or platelet production. However, our data also highlight potential hematologic side-effects of E2 and E2+P4 treatments as white blood cells count and bone marrow cell number were reduced and the tail bleeding time increased (superior to 20 minutes in 3 mice). In addition, further studies are needed to determine the impact of these treatments on older mice, although wild type C57BL/6J do not spontaneously develop risk factor of cardiovascular disease with age [26].

In ApoE-deficient mice given a Western diet, Freudenberger et al. demonstrated that long-term treatment with MPA alone or MPA + E2 increased arterial thrombosis following photochemical injury of the right carotid artery, whereas another synthetic progestin, norethisterone acetate, did not affect arterial thrombosis [23,27]. Altogether, these studies suggested that, in mice, the type of progestogen added is of importance in term of impact on thrombosis. Consistently, recent reports showed that VTE risk is greater in women using MPA than in those taking other progestins, whereas micronized progesterone appears safe and has little or no effect on haemostasis [11] [7–9,28]. Importantly, MPA has agonistic actions on glucocorticoid receptor that could play a role in the deleterious effects observed not only in WHI but also in monkey coronary vasomotion [29].

PRs are expressed in many cells of female reproductive tissues (uterus, ovaries, mammary glands) [4]. In the uterus, the antiproliferative response of P4 on E2 signals is mediated by downregulation of ER expression in the stromal compartment. Indeed, it was previously demonstrated that activation of ERα in uterine stromal cells elicits the release of paracrine factors which are required to induce epithelial cells proliferation [30]. Here, we found that ERα and PR are expressed in the aorta but P4 does not regulate ERα mRNA level. Accordingly, P4 has no impact on E2 mediated gene regulation in this tissue.

In women, as reviewed by Modena et al. [31], compared with no treatment, oral but not transdermal E2 regimen causes a rise in prothrombin activation peptide (F1+2), which is a marker for *in vivo* thrombin generation, a decrease in antithrombin III levels and induces (acquired) resistance to activated protein C [32], resulting in an overall switch toward coagulation activation. We showed here that E2 administered by subcutaneous pellet and combined with P4 protected mice from thrombosis independently of functional deficiencies in coagulation and defects in platelet count or platelet production. In order to better understand the mechanisms underlying the protection against thrombosis, we should now investigate the platelet aggregation, adhesion and secretion responses in E2+P4-treated mice. We previously showed that long-term E2 treatment modulates the mouse platelet proteome [15], whether P4 can impact on these changes remains to be established.

In conclusion in our model, P4 administration did not impact the protective effect of E2 on thrombosis in mice, but opposed as expected the E2 effect on structural and functional changes in the uterus and vagina. Future studies are needed to determine the molecular and cellular mechanisms underlying the effects of E2+P4 treatment on thrombosis in mice. Estrogens do not alter the coagulation factors in mouse (15) as they do in women (31, 32). Whether the prothrombotic risk of estrogens in women is determined by the balance between hepatic (prothrombotic) and hematopoietic (antithrombotic) effects, as suggested in mouse, remains to be determined. In this context, it was important to demonstrate in the present study that progesterone does not interfere adversely on this potential protective action of estrogens on the global risk of thrombosis in mouse.

## Supporting information

S1 Fig Schematic representation of the heart. (TIF)

S2 Fig mRNA level of the indicated gene from aorta of control or E2-, P4- and E2+P4- treated mice analyzed after à 3 weeks-treatment by qPCR. Values correspond to the mean +/- SEM. To test the respective roles of each treatment, a one-way ANOVA was performed and a Bonferroni’s multiple comparison test. * t test vs OVX.
(TIF)
