# Supplementary figures and images for: Effect of chronic estradiol plus progesterone treatment on experimental arterial and venous thrombosis in mouse
Source: PLoS One. 2017 May 9;12(5):e0177043. doi: 10.1371/journal.pone.0177043 (PMC5423617; doi:10.1371/journal.pone.0177043)

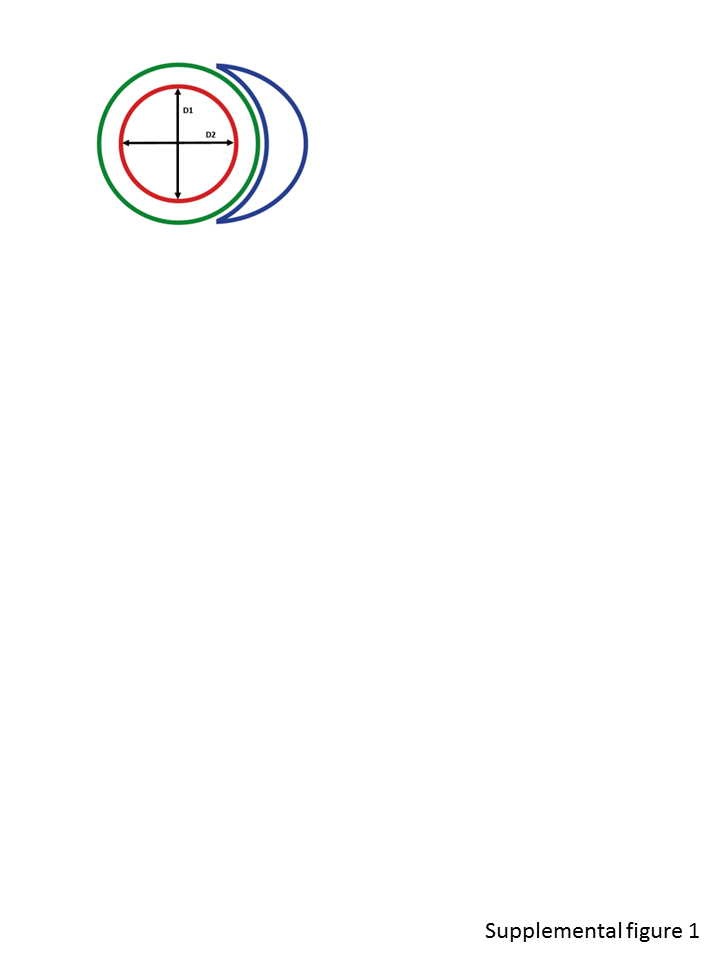

Supplement: S1 Fig — (TIF) [file pone.0177043.s001.tif]

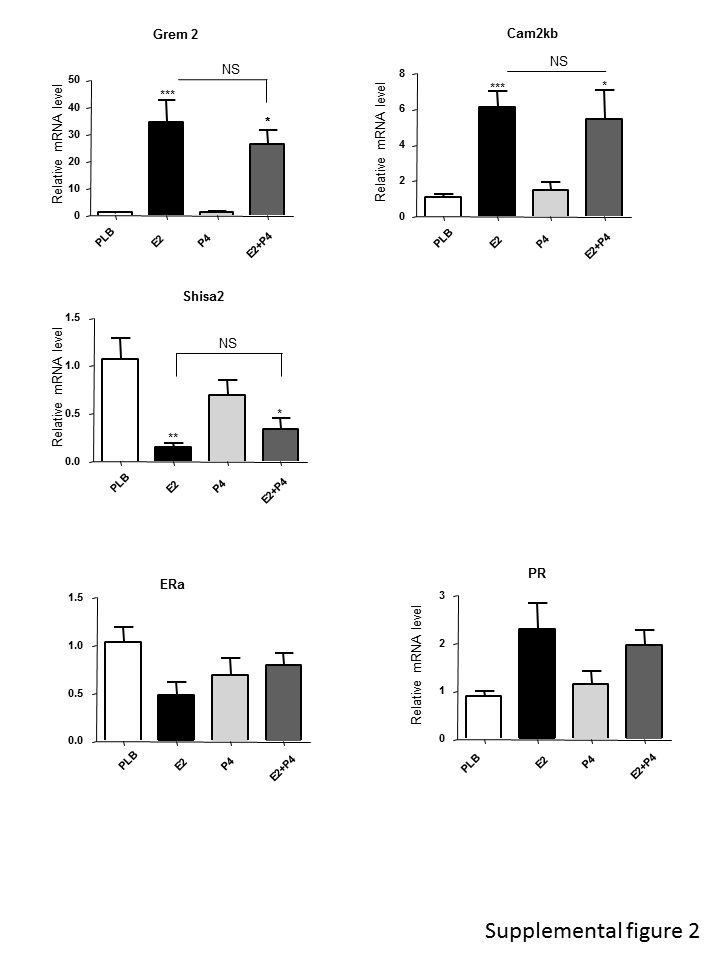

Supplement: S2 Fig — Values correspond to the mean +/- SEM. To test the respective roles of each treatment, a one-way ANOVA was performed and a Bonferroni’s multiple comparison test. * t test vs OVX. (TIF) [file pone.0177043.s002.tif]
